# Serum hyaluronic acid in critically ill dogs and influence of intravenous fluid therapy

**DOI:** 10.1371/journal.pone.0325809

**Published:** 2025-06-13

**Authors:** Manon Rigot, Alexa M. Bersenas, Shane W. Bateman, Shauna L. Blois, Gabrielle Monteith, R. Darren Wood

**Affiliations:** 1 Department of Clinical Studies, Ontario Veterinary College, University of Guelph, Guelph, Ontario, Canada; 2 Department of Pathobiology, Ontario Veterinary College, University of Guelph, Guelph, Ontario, Canada; Gifu University School of Medicine Graduate School of Medicine: Gifu Daigaku Igakubu Daigakuin Igakukei Kenkyuka, JAPAN

## Abstract

**Background:**

The endothelial glycocalyx (EG) appears to play a critical role in physiological vasculo-endothelial function. Sepsis, trauma, and hemorrhagic shock are associated with EG shedding and intravenous fluids have the potential to worsen EG degradation. There is little available research evaluating the relationship between intravenous fluids, inflammation, and EG degradation in critically ill dogs.

**Objective:**

To study EG degradation in critically ill dogs over their first 48 hours of hospitalization and characterize the influence of intravenous fluids and inflammation.

**Methods:**

Hyaluronic acid (HA), a biomarker of EG degradation, was measured in dogs with non-pulmonary sepsis, pulmonary sepsis, or spontaneous hemoperitoneum at five pre-defined time points over 48 hours. The concentration of HA was trended over time, compared between groups, and studied for associations with the cumulative volume of intravenous fluids administered, a pro-inflammatory cytokine (interleukin-6, IL-6), and a biomarker of hypervolemia (atrial natriuretic peptide, ANP).

**Results:**

Concentration of HA was not significantly different between the groups at each time point. It increased over the first 24 hours of the study before reaching a plateau in patients with sepsis and spontaneous hemoperitoneum. Concentration of IL-6 had a significant positive association with HA concentration on presentation in all groups (p = 0.026). Cumulative fluid volume had a significant association with HA concentration during hospitalization in all groups (p = 0.0002). There was no significant effect of ANP on HA concentration. Concentration of HA was associated with disease severity but not with outcome.

**Conclusions:**

In the dogs studied, markers of inflammation and administration of larger volumes of intravenous fluids were associated with increasing HA concentration, and thus presumptive EG degradation. Further research is needed to explore the clinical impact of intravenous fluid therapy on the EG. These findings should be considered carefully by clinicians prescribing fluid resuscitation for critically ill dogs.

## Introduction

The endothelial glycocalyx (EG) is a mesh-like layer which covers the inner surface of blood vessels, positioned at the interface between the bloodstream and endothelial cells [1,2]. It plays a crucial role in regulation of transvascular fluid flux, inflammation, hemostasis, and vascular tone [3–5]. It prevents fluid extravasation via the filter role of its component glycosaminoglycans (GAGs) and the support of a low oncotic pressure in the sub-endothelial space, following the revised Starling’s theory [4,6,7]. It also acts as a dynamic barrier between the leukocytes, platelets, red blood cells, and the active endothelial surface [4,5,8].

Evaluation of EG degradation in vivo is possible using its shed components as biomarkers. These biomarkers have shown good correlation with other structural and functional indicators of EG degradation, and with severity of disease and outcome in people [9–12]. Hyaluronic acid or hyaluronan (HA) is a GAG found in the EG and other extracellular matrices. Quantification of HA concentration in canine blood using commercial human enzyme-linked immunosorbent assay (ELISA) kits has been previously validated and used in research [13–19].

Sepsis, hemorrhagic shock, and trauma are major causes of EG degradation, as demonstrated in both experimental animal models and in people [1,20–23]. Similarly, increased concentrations of HA have been found in dogs with sepsis and hemorrhagic shock [14–16,24]. Evidence that intravenous (IV) crystalloid administration has the potential to be associated with EG degradation has also been demonstrated in experimental animal models [10,25,26], and in people [27–32] and dogs [14,15,17] receiving IV fluids for sepsis, hemorrhagic shock, or surgery. Resuscitation with non-crystalloid fluids (such as plasma) has been associated with less EG degradation than crystalloids [33]. Although the actual effects of IV crystalloids on the EG remain to be determined, degradation of the EG has been suggested as one of the reasons why liberal fluid therapy can contribute to a negative patient outcome [28,29]. Researchers have speculated that the consequences of EG degradation might include interstitial edema, tissue hypoxia, coagulopathy, and dysregulated systemic inflammation [34–38]. The mechanisms responsible for IV fluid-induced EG degradation are not completely understood. Atrial natriuretic peptide (ANP), which is released by the cardiac atria under mechanical wall stress, has been debated as a potential factor [28,29,31,39–44]. Other hypotheses include direct effect of shear stress on the endothelium [29], and modulation of inflammatory proteins by IV fluids [45,46].

Considering the potential negative effects of IV fluids on the EG, a better understanding of their impact is warranted for future development of evidence-based recommendations on therapeutic fluid prescriptions which could protect the EG. Minimal investigation has been reported in veterinary medicine. Using HA serum concentrations to characterize EG degradation in critically ill dogs, our study aimed to explore the association of HA with inflammatory biomarkers and with cumulative IV fluid volumes during the resuscitation period and beyond to 48 hours. Critically ill dogs with pulmonary sepsis (PS), non-pulmonary sepsis (NPS; e.g., septic peritonitis), and spontaneous hemoperitoneum (HEM) were enrolled. The study was designed based on the hypothesis that these three patient groups would differ in fluid volumes received during resuscitation and ongoing hospitalization, following current recommendations, dogs with PS being expected to receive more conservative IV fluid volumes, and dogs with NPS and HEM likely to be supported with more aggressive IV fluid volumes [47–49]. Based on this, the main objective of our study was to compare HA shedding in dogs with severe inflammation (sepsis groups) vs. dogs with minimal inflammation (HEM) and in dogs with expected similar inflammatory status but differing fluid resuscitation strategies (PS vs. NPS). A secondary objective was to investigate the association of HA with the cumulative volume of IV fluids administered and with biomarkers of hypervolemia (ANP) and inflammation (IL-6). We hypothesized that HA would be higher in patients with higher cumulative fluid volumes, that HA would be lower in dogs with PS (conservative fluid therapy) compared to NPS, and that HA would be lower in dogs with HEM (non-inflammatory) compared to dogs with NPS.

## Materials and methods

### Study design

Patients were enrolled in a prospective observational study conducted in the intensive care unit (ICU) of a veterinary teaching hospital between February 2022 and September 2023. All procedures were approved by the Institutional Animal Ethics Committee (AUP#4422) and informed owner consent was obtained for all dogs.

Dogs were enrolled if they were admitted to the ICU with a diagnosis of sepsis (subdivided into pulmonary sepsis and sepsis of any non-pulmonary cause [e.g., septic peritonitis]) or HEM, and had planned hospitalization expected to last for at least 24 hours. Inclusion criteria for each group are summarized in Table 1. Briefly, inclusion in either sepsis group required at least two of the systemic inflammatory response syndrome (SIRS) criteria [50]. In addition, for the PS group, history consistent with pneumonia and radiographic or ultrasonographic evidence of pneumonia were required. Dogs with fungal pneumonia were excluded. Inclusion in the NPS group required confirmation of an infectious process at admission or within 12 hours of hospitalization. This was based on identification of intracellular bacteria during cytological examination of the source or positive results on bacterial culture. Inclusion in the HEM group was based on evidence of hypovolemic shock and confirmation of hemorrhagic abdominal effusion without evidence of trauma or coagulopathy. Study exclusion criteria included body weight of less than 10 kg to avoid iatrogenic anemia, lack of confirmation of sepsis following initial inclusion in either sepsis group, or if blood collection could not be performed at defined time points for at least the first 24 hours of the study. Dogs were not excluded from the study if only one sample was missed. Dogs with a history of comorbidities were not excluded, however this information was recorded. For each study dog, all decisions regarding medical and surgical interventions, including volume resuscitation, medications, transfusion needs, and diagnostic tests were at the discretion of the attending clinician.

**Table 1:** Inclusion criteria for the three study groups.

Pulmonary sepsis (PS)	Non-pulmonary sepsis (NPS)	Spontaneous hemoperitoneum (HEM)
At least 2 of the following SIRS criteria on presentation: T > 39.4°C or < 37.8°C HR > 140 beats/min RR > 30 breaths/min, pCO2 < 30 mmHg • WBC > 16 or <6 x10^9/L; Bands > 3%	At least 2 of the following SIRS criteria on presentation: T > 39.4°C or < 37.8°C HR > 140 beats/min RR > 30 breaths/min, pCO2 < 30 mmHg WBC > 16 or < 6 x 10^9/L; Bands > 3%	Presence of a spontaneous hemoperitoneum: Hemorrhagic abdominal effusion (PCV > 12%) No history of trauma No primary coagulopathy
Suggestion of pneumonia on thoracic imaging: Cranioventral pulmonary consolidation on thoracic radiographs or CT imaging OR presence of B-lines and/or shred signs with a ventral pattern on point-of-care ultrasound	Confirmation of an infectious process: Observation of overt intestinal leakage or necrotic bowel during surgery (septic peritonitis) OR presence of at least 1 of the following criteria: positive results on bacterial culture of the source identification of intracellular bacteria during cytologic examination of the source	Evidence of cardiovascular decompensation on presentation: Heart rate > 140 beats/minute OR respiratory rate > 40 breaths/minute OR mean arterial pressure < 75 mmHg OR systolic blood pressure < 90 mmHg OR lactate > 2 mmol/L

A previous publication was used for sample size determination [14]. Based on the mean difference and variance in HA in a group of dogs hospitalized with septic peritonitis, the number of dogs required to detect a change in HA of 30 ng/mL between groups with a power of 80%, was 18 dogs per group using a paired t-test with a p-value of < 0.05.

### Clinical data collection

Patient signalment and weight were recorded, as well as known comorbidities that might affect HA concentration, including liver disease, diabetes mellitus, malignancy, and renal disease. Data recorded at admission included clinical and laboratory findings: mentation, rectal temperature, heart rate, respiratory rate, systolic and mean blood pressure, oxygen saturation (SpO2), packed cell volume (PCV), total proteins (TP), blood glucose, blood lactate, venous partial pressure of CO2 (pCO2), complete blood count and serum biochemistry data, and presence of cavitary effusion based on point of care ultrasound. Illness severity score at the time of admission was calculated using the Acute Patient Physiological and Laboratory Evaluation (APPLE) score (APPLE_full_ and APPLE_fast_) [51]. Results of cytology, bacterial culture, histology, and point-of-care effusion analysis (PCV, glucose, lactate) were also recorded when performed. Anesthetic records of dogs that underwent surgery during the study period were reviewed for time and duration of surgery.

The volume and type of IV fluids (isotonic or hypertonic crystalloids, synthetic colloids, and blood products) administered during the study, including fluids administered in surgery, were retrieved from the patients’ medical and anesthetic records. Total drug volumes administered as a continuous rate infusion exceeding 0.25 mL/kg/h were also recorded throughout the study period. Cumulative fluid volume was calculated for each patient at each blood collection timepoint. Fluids administered before presentation to our hospital were also recorded as much as possible but were not included in the total fluid volume due to inability to report the precise volume and time course for many of these patients.

The visit outcome (alive at discharge, dead, or euthanized) was recorded for all dogs. For euthanized patients, the reason for euthanasia was categorized as financial or due to poor prognosis.

### Sample collection and biomarker measurement

Blood (3 mL) was collected from each dog at predefined time points: on presentation (T0), after 2 + /- 1 hours (T2), after 6 + /- 1 hours (T6), after 24 + /- 12 hours (T24), and after 48 + /-12 hours (T48). The initial sample was collected during placement of an IV catheter on admission to the hospital, prior to fluid administration. Subsequent samples were collected via an indwelling sampling catheter (jugular catheter or arterial catheter) if available, or via jugular or saphenous venipuncture. Timepoints were set such that the T2 sample would be collected prior to surgery and T6 would be collected post-operatively for most of the enrolled dogs where surgical intervention was indicated (e.g., hemoperitoneum, septic peritonitis). Samples for T24 and T48 were collected at times when other blood samples were requested by the attending clinician to reduce need for repeat venipuncture.

Blood was collected in a vacuum-sealed plastic tube without additives. Whole blood was allowed to clot for 2 hours at room temperature before being centrifuged at 1000xg for 30 minutes. The serum was separated, aliquoted into microcentrifuge tubes, and stored at −80°C for later batch analysis.

Biomarker measurements were conducted at a different site (The Ohio State University). All samples were transported via overnight courier on dry ice. Upon arrival the frozen samples were transferred into a −80°C freezer until later batch analysis. Biomarker measurements were performed using commercial ELISA kits validated for use in dogs following manufacturer’s instructions. The kits used included HA (QuantikineTM ELISA Hyaluronan Immunoassay, R&D Systems, Minneapolis, MN, United States), ANP (ANP BioAssayTM ELISA kit (canine), US Biological, Salem, MA, United States), and inflammatory cytokine IL-6 (QuantikineTM ELISA IL-6, R&D Systems, Minneapolis, MN, United States). All measurements were performed in duplicate. Samples were diluted if the measured biomarker concentration was above the limit of detection of the assay. The assay’s lower limits of detection reported by the manufacturer were 0.068 ng/mL for HA, 0.031 ng/mL for IL-6, and 0.012 ng/mL for ANP.

### Statistical methods

Data were checked for normality by examination of the residuals, quantile-quantile plots and normality tests that included the Shapiro-Wilk test, Kolmogorov-Smirnov test, Cramer-von Mises test, and Anderson-Darling test. When necessary, data was log-transformed for analysis to meet the assumption of normality. Data was normally distributed after log-transformation. For presentation of means and 95% confidence intervals, the data was back-transformed. For repeated measures, Akaike’s information criterion was used to determine the best covariance structure.

To compare the volume of fluid administered in the different groups, the cumulative fluid volume was computed for each animal at each time point and the means compared between groups using an ANOVA for repeated measures. The total cumulative fluid volumes and total cumulative fluid volumes divided by time were also compared between groups using an ANOVA. Post-hoc Tukey test was applied when the overall f test for the group was significant. An ANOVA and post-hoc Tukey test were also used to compare the concentrations of HA, IL-6, and ANP between the three groups at each time point and an ANOVA for repeated measures was used to compare HA concentrations between time points.

A Pearson correlation test was used to test for an association between ANP concentration and cumulative fluid volumes administered during the study.

To investigate possible predictors of HA concentration at T0, a general linear model was built. Parameters included group (HEM, NPS, and PS), the presence of malignancy as a comorbidity, the administration of IV fluids before hospital admission, and T0 APPLE_fast_ score, IL-6 and ANP concentrations. APPLE_fast_ was used over APPLE_full_ due to fewer missing data for their respective calculations. This analysis determined covariates of HA at T0. Then, to investigate determinants of HA concentration during hospitalization (after presentation), a mixed effect linear model for repeated measures was applied to the data for time points starting at T2. Possible explanatory variables included HA concentration at T0 and its covariates determined in the previous analysis. Fixed effects of time, surgery, cumulative fluid volume administered during the study time (over 48 hours for all dogs except 7 dogs who were only included in the study for 24 hours), and IL-6 and ANP concentrations were modelled, including interactions. Time was included in the model as a continuous variable with the exact time of sampling rather than the predefined time points where variation was accepted a priori. Quadratics for continuous parameters and interactions were also included in the models. To account for repeated measures, the first order autoregressive AR(1) was the best fit for the covariance structure. The random effect of animal (within group) gave the lowest Akaike’s information criterion when compared to other tested correlation structures. Models were reduced by removing non-significant effects (p > 0.05).

A logistic regression was used to determine if HA at T0, maximal HA concentration, and the difference between HA at T0 and HA at T24 were predictors of outcome.

A commercial software (SAS/STAT® 9.4, SAS Institute, Cary, NC) was used for statistical analysis.

## Results

### Patient characteristics

**Fig 1:**
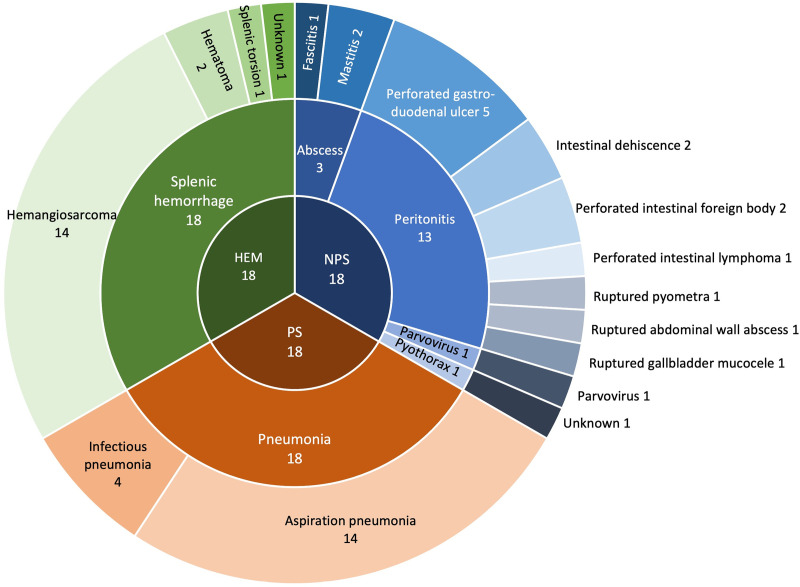
Etiology and final diagnosis for dogs in each group. SH: spontaneous hemoperitoneum, NPS: non-pulmonary sepsis, PS: pulmonary sepsis.

Fifty-five dogs were enrolled in the study. One dog was later excluded from analysis due to two missed samples, leaving 54 dogs included (18 per group). Most dogs were mixed breed dogs (11), and among pure breed dogs, Labrador Retrievers (7) and Golden Retrievers (6) were the most represented. Other breeds included German Shepherd (3), Great Dane (3), Bernese Mountain Dog (2), Cane Corso (2), English Bulldog (2), Mastiff (2), Portuguese Water Dog (2), Standard Poodle (2), Airedale Terrier (1), Australian Shepherd (1), Basset Hound (1), English Sheepdog (1), Flat Coated Retriever (1), Rottweiler (1), Schnauzer (1), Shetland Sheepdog (1), Springer Spaniel (1), Vizsla (1), and Weimaraner (2). There were 24 neutered males, 19 spayed females, 8 intact males and 3 intact females. Severity of illness and comorbidities for dogs in each group are presented in Table 2. Based on available data, APPLE_full_ and APPLE_fast_ were calculated for 47/54 and 51/54 dogs, respectively. Septic shock was diagnosed in 4 patients based on the need for vasopressors to maintain mean arterial pressure (MAP) ≥ 65 mmHg. There were eight patients with hyperglycemia where blood glucose measured between 8–16 mmol/L at one or two timepoints of the study (4 in the NPS group and 4 in the HEM group). Etiology and final diagnosis for dogs in each group are summarized in Fig 1. Final diagnosis could not be determined for one dog in the HEM group due to technical issues with histology. An underlying cause could not be determined for the dog with pyothorax.

**Table 2:** Signalment, severity of illness, comorbidities, timing of surgery, types and volumes of intravenous fluids received throughout the study, and outcome for patients in each group.

		Spontaneous hemoperitoneum (HEM) (n = 18)	Non-pulmonary sepsis (NPS) (n = 18)	Pulmonary sepsis (PS) (n = 18)	Total (n = 54)
Signalment	Age (years)	8.4 (7.3-10.1)	3.0 (2.0-6.2)	3.4 (2.4-8.5)	6.6 (2.5-9.1)
	Weight (kg)	33.0 (28.8-39.7)	31.5 (29.0-39.3)	32.9 (23.4-37.3)	32.6 (27.2-39.0)
Severity of illness	APPLE_full_ score^a^	35.0 + /- 7.7	36.5 + /- 12.0	31.4 + /- 9.0	33.8 + /- 10.1
	APPLE_fast_ score^b^	27.7 + /- 8.0	29.3 + /- 5.6	22.3 + /- 4.7	26.4 + /- 6.9
	Septic shock	0 (0)	3 (16.7%)	1 (5.6%)	4 (7.4%)
Comorbidities	Malignancy	15 (83.3%)	2 (11.1%)	3 (16.7%)	20 (37.0%)
	Hyperglycemia	4 (7.4%)	4 (7.4%)	0 (0)	8 (14.8%)
	Liver disease	0 (0)	1 (5.6%)	0 (0)	1 (1.9%)
Surgery performed (time after presentation)	2-6 hours	17 (94.4%)	13 (72.2%)	0 (0)	30 (55.6%)
	6-12 hours	1 (5.6%)	1 (5.6%)	0 (0)	2 (3.7%)
	30 hours	0 (0)	1 (5.6%)	0 (0)	1 (1.9%)
	No surgery	0 (0)	3 (16.7%)	18 (100%)	21 (38.9%)
Fluids received pre-referral	Yes	5 (27.8%)	9 (50%)	7 (38.9%)	21 (38.9%)
	No	13 (72.2%)	9 (50%)	11 (61.1%)	33 (61.1%)
Types of intravenous fluid products received during the study	Isotonic crystalloids	18 (100%)	18 (100%)	18 (100%)	54 (100%)
	Hypertonic crystalloids	4 (22.2%)	7 (38.9%)	1 (5.6%)	12 (22.25)
	Synthetic colloids	1 (5.6%)	0 (0)	0 (0)	1 (1.9%)
	Packed red blood cells	11 (61.1%)	0 (0)	0 (0)	11 (20.3%)
	Whole blood	2 (11.1%)	0 (0)	0 (0)	2 (3.7%)
	Fresh Frozen Plasma	1 (5.6%)	7 (38.9%)	0 (0)	8 (14.8%)
Cumulative fluid volume^c^	Cumulative volume at 24 hours (mL/kg)	97.1 (85.3–117.4)	119.0 (91.3–170.5)	54.1 (39.1–61.2)	87.6 (57.8-119.3)
	Total cumulative volume during study (mL/kg)^c^	129.5 (98.8–154.7)^θ*^	183.2 (136.3-230.4)^*^	86.2 (69.9–116.5)^θ^	127.8 (91.6-162.5)
	Total cumulative volume divided by study duration (mL/kg/h)	3.8 (2.7–4.7)^θ^	4.0 (2.9-6.0)^*^	2.1 (1.7–2.6)^θ *^	3.1 (2.3-4.7)
	Rate of fluids administered between 0 and 24 hours (mL/kg/h)	5.1 (3.6-7.6)	5.6 (4.7-8.7)	2.2 (1.8-2.7)	4.7 (2.5-7.1)
	Rate of fluids administered between 24 and 48 hours (mL/kg/h)	1.7 (1.4-2.1)	2.0 (1.5-4.0)	1.7 (1.4-2.4)	1.8 (1.5-2.7)
Outcome	Dead	0 (0)	1 (5.6%)	0 (0)	1 (1.9%)
	Alive at discharge	18 (100%)	15 (83.3%)	13 (72.2%)	46 (85.2%)
	Euthanized (prognosis)	0 (0)	2 (11.1%)	4 (22.2%)	6 (11.1%)
	Euthanized (financial)	0 (0)	0 (0)	1 (5.6%)	1 (1.9%)
Duration of hospitalization (days)	Alive at discharge	2.0 (1.3-2.0)	5.0 (3-6.5)	3.0 (3.0-4.0)	3.0 (2.0-4.5)
	Dead or euthanized	–	3.0 (3.0-3.5)	3.0 (2.0-4.0)	3.0 (2.8-4.0)
	Whole population	2.0 (1.3-2.0)	4.0 (3.0-5.0)	3.0 (2.3-4.0)	3.0 (2.0-4.0)

Data are presented as number (%) of dogs, median (Q1-Q3), or mean + /- SD.

^a^APPLE_full_ score calculated on 13 patients with spontaneous hemoperitoneum, 18 patients with pulmonary sepsis, and 16 patients with non-pulmonary sepsis.

^b^APPLE_fast_ score calculated on 17 patients with spontaneous hemoperitoneum, 18 patients with pulmonary sepsis, and 16 patients with non-pulmonary sepsis.

^c^Cumulative fluid volumes were calculated over the study period. In patients who were no longer enrolled in the study at the last time point T48 (5 discharged, 2 deceased), total cumulative fluid volumes were calculated over 24 hours (until the fourth time point T24).

Total cumulative fluid volumes divided by study duration are also presented to account for the effect of dogs removed before the last time point (T48) sampling.

θ, * Statistically significant difference between the volumes (p < 0.05).

Data was recorded and samples collected for all time points except the T48 time point in 7 dogs because 5 dogs with HEM had been discharged and 2 dogs with PS were deceased.

### Therapy provided

**Fig 2:**
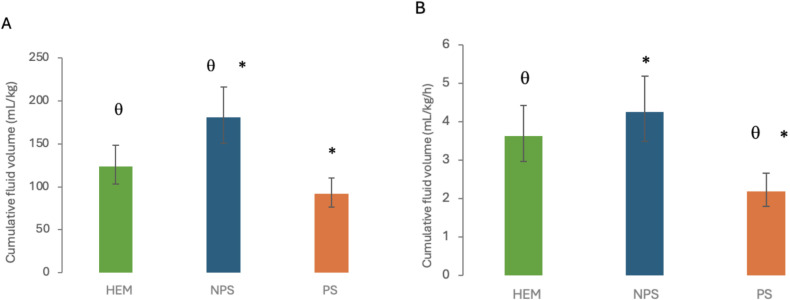
Cumulative fluid volumes in each group: total cumulative fluid volumes (A) and total cumulative fluid volumes divided by duration of inclusion in the study (B). *, θ: Statistically significant difference (p < 0.05) between the corresponding two groups. HEM: spontaneous hemoperitoneum, NPS: non-pulmonary sepsis, PS: pulmonary sepsis.

In 21 dogs, IV fluids had been administered before referral to our hospital. In-hospital fluid administration consisted of isotonic balanced crystalloids (Plasmalyte-A, Baxter), hypertonic saline solution (5% Sodium Chloride, B Braun), synthetic colloids (6% hydroxyethyl starch 130/0.4, Voluven, Fresenius Kabi), and blood products (packed red blood cells, whole blood and fresh frozen or stored plasma). Fluid types and total cumulative volumes in each group are presented in Table 2. Total cumulative fluid volume was calculated at T48 for all dogs except those who were no longer enrolled in the study at T48 (5 discharged, 2 deceased), for which total cumulative fluid volumes were calculated over 24 hours (at T24). Based on the ANOVA, patients in the NPS group received significantly more fluids than patients in the PS group (p < 0.0001), and than patients in the HEM group (p = 0.013) (Fig 2). The cumulative fluid volume was higher in dogs with HEM than dogs with PS, however this difference did not reach statistical significance (p = 0.056). To account for the fact that 5 dogs with HEM and 2 dogs with PS were discharged or deceased, respectively, at T48, and that their total cumulative fluid volumes were calculated over 24 hours rather than 48 hours, the analysis was also repeated after dividing the total cumulative fluid volume by the duration of inclusion in the study for each dog (Table 2). This cumulative fluid volume divided by time was independent of study duration for each dog. It was still significantly higher in NPS than PS (p < 0.0001), but it was also significantly higher in HEM than PS (p = 0.0019) and there was no significant difference between NPS and HEM (p = 0.492).

Surgical intervention was required during the study time in 33/54 dogs. Timing of surgery is presented in Table 2. Three dogs with NPS were managed medically: a dog with parvoviral enteritis, a dog with mastitis and a dog with fasciitis. Surgery due to NPS was delayed beyond the 6-hour sampling time point in one dog with pyothorax where surgery was performed 12 hours after presentation, and in another dog with septic peritonitis initially operated at the referring veterinarian where intestinal dehiscence was noted 30 hours after presentation with subsequent emergency surgical revision at our hospital.

### Biomarkers

All samples were successfully collected except for those dogs no longer in the study at T48 (7/54 dogs). These dogs were still included in the analysis for the remaining sample times.

**Fig 3:**
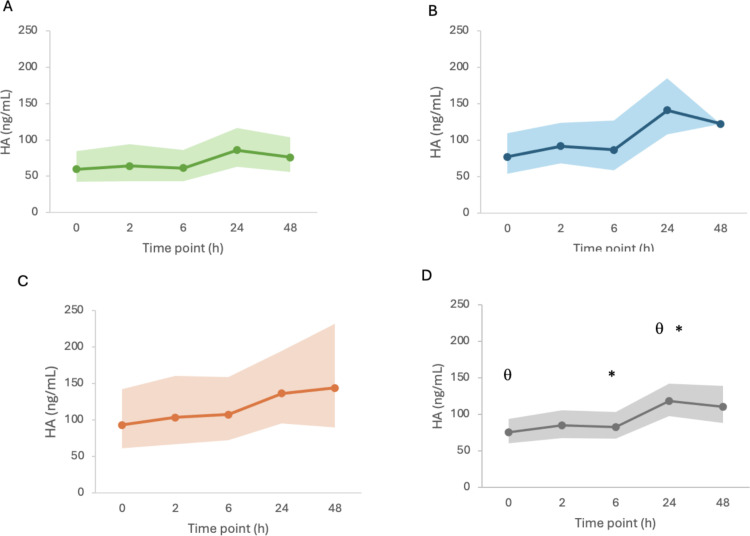
Mean hyaluronic acid concentration in time in patients with spontaneous hemoperitoneum (A), non-pulmonary sepsis (B), pulmonary sepsis (C), and all groups combined (D). The bolded line represents the mean value of hyaluronic acid and the underlying shadow represents the 95% confidence interval. *, θ: Statistically significant difference (p < 0.05) between the corresponding time points.

The progression of mean HA in time was plotted for all patients (Fig 3). Overall, HA concentration was significantly higher at T24 than at T0 (p = 0.010) and at T6 (p = 0.003) for all groups combined.

When using an ANOVA, HA concentration was not significantly different between groups at any time point (Table 3), which refuted our primary hypothesis. The overall mean HA for all time points was significantly lower in PS than in NPS (p = 0.021) but there were no statistically significant differences between HEM and NPS or between HEM and PS. IL-6 concentration was significantly higher in NPS than HEM (p = 0.003) and tended to be higher in NPS than PS (p = 0.052) (Table 4). There were no significant differences in ANP concentration between the groups at any time point and no association between group and ANP concentration (p = 0.1360). There was no correlation of ANP with cumulative fluid volume (p = 0.362).

**Table 3:** Mean hyaluronic acid concentration in each group at each time point.

	HEM	NPS	PS	p-value NPS vs. HEM	p-value NPS vs. PS	p-value HEM vs. PS
T0	77.2 (53.4-111.5)	93.3 (64.6-134.7)	59.7 (41.3-86.2)	0.474	0.092	0.330
T2	92.1 (63.6-133.0)	103.5 (71.7-149.6)	64.0 (44.3-92.4)	0.656	0.070	0.169
T6	86.6 (60.0-125.1)	107.4 (74.4-155.1)	61.2 (42.3-88.3)	0.415	0.123	0.188
T24	141.2 (97.6-203.7)	136.3 (94.4-196.9)	85.8 (59.4-123.9)	0.897	0.080	0.060
T48	115.5 (76.6-174.1)	144.0 (99.7-208.0)	74.3 (50.7-108.9)	0.429	0.095	0.123
Overall	100.1 (76.4-131.1)	115.3 (88.2-150.7)	68.3 (52.2-89.4)	0.738	**0.021**	0.120

T0: on presentation to the hospital, T2: 2 + /- 1 hours after presentation, T6: 6 + /- 1 hours after presentation, T24: 24 + /- 12 hours after presentation, T48: 48 + /-12 hours after presentation.

HEM: spontaneous hemoperitoneum, NPS: non-pulmonary sepsis, PS: pulmonary sepsis.

Data is presented as mean (95% confidence interval).

Concentrations are in ng/mL. The bolded p-value is < 0.05.

**Table 4:** Mean IL-6 concentration in each group at each time point.

	HEM	NPS	PS	p-value NPS vs. HEM	p-value NPS vs. PS	p-value HEM vs. PS
T0	71.3 (34.3-148.0)	399.0 (192.2-828.5)	191.8 (92.4-398.2)	**0.012**	0.163	0.160
T2	72.0 (34.6-149.4)	481.8 (232.0-1000.2)	193.7 (93.3-402.2)	**0.003**	0.191	0.129
T6	106.3 (51.2-220.7)	658.9 (317.4-1368.1)	153.3 (73.8-318.2)	**0.004**	**0.025**	0.720
T24	68.5 (33.0-142.3)	137.4 (66.2-285.3)	106.5 (51.3-221.1)	0.291	1.000	0.600
T48	36.8 (16.9-80.2)	81.0 (39.1-168.2)	29.4 (13.9-62.0)	0.380	0.137	1.000
Overall	67.2 (36.0-125.7)	269.1 (144.3-501.9)	112.2 (60.1-209.5)	**0.003**	0.052	0.250

T0: on presentation to the hospital, T2: 2 + /- 1 hours after presentation, T6: 6 + /- 1 hours after presentation, T24: 24 + /- 12 hours after presentation, T48: 48 + /-12 hours after presentation.

IL-6: interleukin-6, HEM: spontaneous hemoperitoneum, NPS: non-pulmonary sepsis, PS: pulmonary sepsis.

Data is presented as mean (95% confidence interval).

Concentrations are in pg/mL. The bolded p-values are < 0.05.

**Fig 4:**
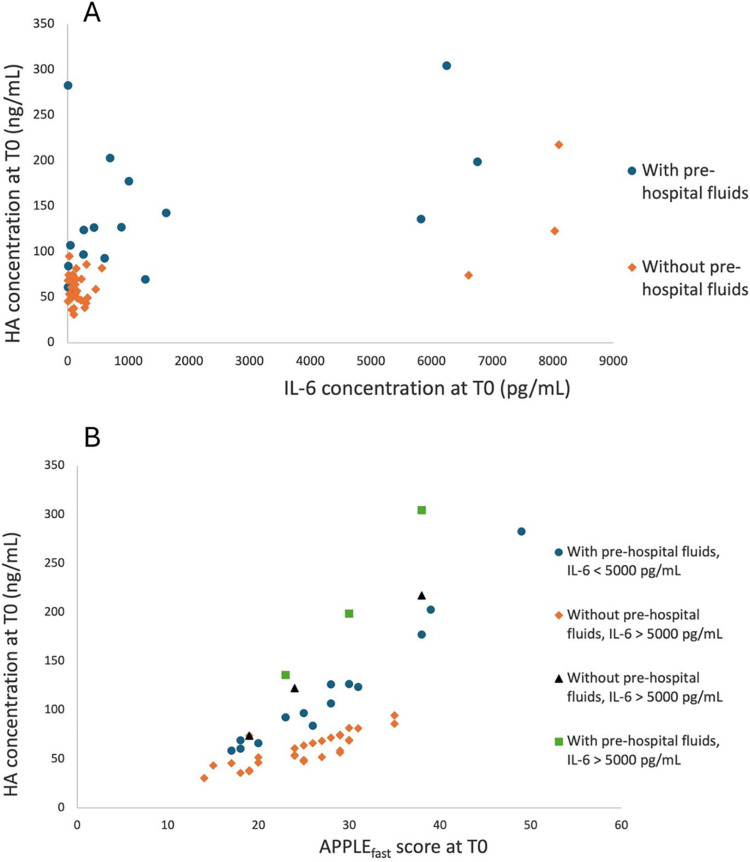
Predicted mean hyaluronic acid concentration at T0 based on IL-6 concentration at T0 (A) and APPLE_fast_ score at T0 (B). HA concentrations were back-transformed from the logarithmic transformation. The concentration of IL-6, the APPLE_fast_ score, and the administration of IV fluids before hospital admission have a significant positive effect on HA concentration at T0 (p = 0.026, p = 0.015, and p = 0.015, respectively). HA: hyaluronic acid, IL-6: interleukin-6.

In order to investigate factors having a significant effect on HA at T0, a mixed model was built. The group, the presence of malignancy, and ANP concentration were not found to have a significant effect on HA at T0 and were excluded from the model. The concentration of IL-6 and APPLE_fast_ score had a significant positive effect on HA concentration at T0 (p = 0.026 and p = 0.015, respectively) (Fig 4). Patients who had received IV fluids before hospital admission had a higher HA concentration at T0 (p = 0.015) than those not having received IV fluids before. The concentration of IL-6 at T0, the APPLE_fast_ score at T0, and the administration of IV fluids before admission were therefore independent predictors of HA concentration at T0.

**Fig 5:**
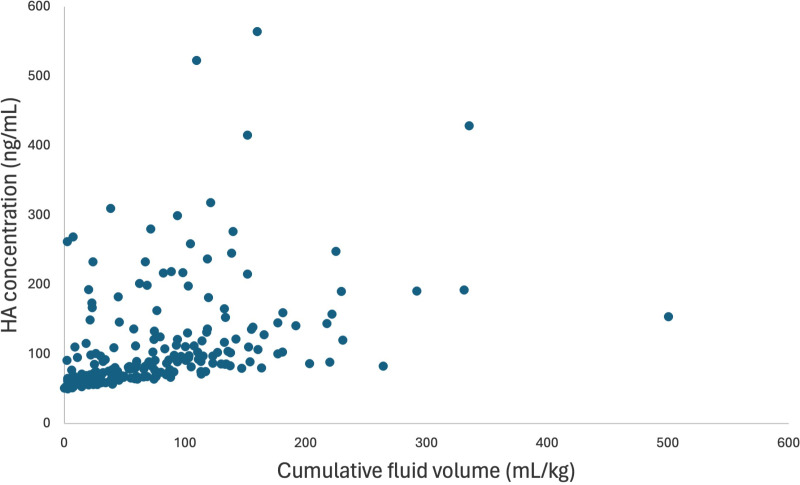
Predicted mean hyaluronic acid as a function of cumulative fluid volume for time points T2 to T48. HA concentrations were back-transformed from the logarithmic transformation. The cumulative fluid volume has a significant positive effect on HA from T2 to T48 (p = 0.0002). HA = hyaluronic acid.

To investigate the factors having an effect on HA concentration during hospitalization, beyond T0, a second mixed model was built, including HA concentration at T0 as well as its previously listed covariates, time, the cumulative volume of intravenous fluids administered in hospital, and IL-6 and ANP concentrations during hospitalization (starting at T2). In this model, when accounting for HA concentration at T0, the effect of the covariates: group, the administration of IV fluids before hospital admission, the presence of malignancy, IL-6 and ANP concentrations at T0 were not significant and they were removed from the model. The model identified that the cumulative volume of fluids had a significant positive effect on HA (p = 0.0002) (Fig 5). This effect did not change among groups. The concentration of ANP and IL-6 during hospitalization, time, and group did not have a significant effect on HA concentration during hospitalization.

### Outcome

Forty-six dogs (85%) survived to discharge. Outcome and duration of hospitalization for dogs in each group are presented in Table 2. Among the patients with septic shock, 3 out of 4 did not survive to discharge.

Hyaluronic acid concentration at T0, maximum HA concentration, and the difference in HA concentration between T0 and T24 were not significant predictors of outcome based on a logistic regression (p = 0.258, p = 0.503, and p = 0.393, respectively).

## Discussion

This study was designed to investigate potential associations between IV fluid administration, inflammation, and EG degradation. In order to study these factors, predefined groups of dogs were selected to reflect anticipated differing delivered IV fluid volumes during the resuscitative and early hospitalization period (conservative in PS and more liberal in NPS and HEM). Similarly, groups were also selected to compare and contrast variable degrees of inflammation (less inflammation anticipated in HEM and more inflammation in NPS and PS). The study results demonstrate that, in our hospital, dogs with PS received significantly less IV fluids over the duration of the study than dogs with HEM and NPS. Our hypothesis was also met in that dogs with HEM had lower inflammatory cytokine IL-6 concentrations than dogs with NPS, although there was no statistical difference in IL-6 concentration between dogs with HEM and dogs with PS. The distribution of IL-6 concentration in patients with PS was very wide, reflecting heterogeneous inflammation in this group and likely explaining the lack of statistically significant difference with the HEM group. Despite these differences in both volume of IV fluids administered and inflammation, no significant difference in HA concentration was identified between the groups at any time point when assessed by simple ANOVA. When looking at overall HA concentration including all time points, HA concentrations were significantly higher in dogs with NPS than with PS. This finding may be attributed to larger volumes of IV fluids administered to the NPS group or to more severe inflammation in the NPS group than the PS group based on higher IL-6 concentrations, although only statistically significant at T6.

The lack of significant difference in HA concentration between groups at each time point may be explained by the smaller number of samples available when looking at separate time points, however other factors could also have been involved. Since this is an observational study, several parameters were likely at play both between and within groups, potentially affecting HA concentrations, which were investigated in mixed models. This study identified some interesting findings based on the mixed model analyses. First, APPLE_fast_ score and IL-6 concentration at T0, along with the administration of IV fluids before hospitalization, were identified to have a significant effect on HA concentration at T0. Thereafter, during hospitalization (starting at T2), HA concentration was determined by its concentration at T0 and the cumulative volume of IV fluids administered during. Thus, the overall findings of these analyses were that HA concentration was determined by the inflammatory status (IL-6 concentration), the severity of illness (APPLE_fast_ score), and the administration of IV fluids both before hospitalization and during hospitalization, for which the effect was volume-dependent. The effects of the degree of inflammation and disease severity were most predominant on presentation. This could indicate that, although patients were presented with a certain degree of EG degradation caused by the disease process, ongoing EG degradation may have been caused by IV fluids administered in hospital. The disease group did not have a significant effect on HA concentration, which could be explained by the fact that levels of inflammation, disease severity, and administered volumes of IV fluids were heterogeneous within groups.

Interestingly, HA concentration in the NPS group was not significantly different from HA concentration in dogs with HEM, indicating that patients with hemorrhagic shock might have a similar degree of endotheliopathy as patients with sepsis, although evaluated with a single biomarker in this study. Biomarkers of EG degradation have repeatedly been reported to be higher in patients with sepsis than in healthy controls and patients with simple infection [12,30,52,53]. However, a comparison between patients with sepsis and patients with hemorrhagic shock is not currently available in the human medical literature. Studies evaluating people with severe trauma and experimental rodent models demonstrate that patients with hemorrhagic shock can have markedly elevated biomarkers of EG degradation as well [10,20,22,23,54–56]. In the veterinary literature, HA, as a biomarker, has only been explored in hemorrhagic shock models in healthy dogs with experimentally induced hemorrhage. Results are contradictory, with one study showing an increase in HA following 60 minutes of hemorrhagic shock [15] and another failing to show an increase in HA following 10 minutes of hemorrhagic shock [57].

Our study also aimed to explore the pattern of HA biomarker concentrations in dogs with systemic illness and to investigate the timing of peak HA concentration based on predefined sampling times. In our study, HA concentration increased progressively from T0 to T24 in patients with NPS and HEM, before reaching a plateau. The differences were only statistically significant between T24 and T0, and T24 and T6. The concentration of HA at T48 was more variable in NPS, which is consistent with the heterogenous progression of disease in this group compared to the other two groups. Unfortunately, due to the study design, no data was available beyond 48 hours. In previous human studies, different EG biomarkers were found to have different variations over time [12,53] and HA did not change significantly over 4 days in 8 dogs with septic peritonitis [14]. Therefore, expected changes in HA are non-predictable when reviewing the human and veterinary literature and may be related to the individual patient’s disease progression. The increase in HA concentrations over the first 24 hours in the NPS and HEM groups could be due to initial fluid resuscitation, with de-escalation of IV fluids progressing into the second day of hospitalization. This would be supported by the difference in the rate of fluid administration during the first day and the second day of the study.

The effect of IV fluid volumes on EG degradation has been documented in people, with an increase in EG biomarkers (HA, heparan sulfate) associated with the volume of fluids delivered during resuscitation in septic patients [29,30,58]. However, in other human studies, the volume of fluids administered did not seem to have an effect on EG biomarker concentrations and the effect of IV fluids on the EG should therefore be interpreted cautiously, especially in patients with sepsis [27,59–62]. Studies in veterinary medicine exploring the effects of IV fluids on the EG are limited. A study conducted in healthy dogs undergoing elective surgery reported an increase in HA following anesthetic IV fluid administration (at 5 mL/kg/hr and 10 mL/kg/h) [17] and a retrospective case series of 8 dogs with septic peritonitis followed daily during their recovery reported a significant effect of cumulative IV fluid volume on HA concentration when accounting for IL-6 concentration [63]. In an experimental model of canine hemorrhagic shock, resuscitation with high volumes of crystalloids (80 mL/kg) was also associated with an increase in HA concentration [15]. To date, our results are consistent with previous findings that IV fluid therapy may have an effect on the EG.

Interleukin-6 is a pro-inflammatory cytokine which is a well-known diagnostic and prognostic marker of sepsis in people [64,65] and dogs [14,66–68]. Our study identified higher IL-6 concentrations in sepsis, most notably of non-pulmonary origin, as well as a positive effect of IL-6 on HA at T0 in all groups. Interleukin-6 has been reported to be positively correlated with EG biomarkers in people with sepsis and trauma [52,69,70] and was a significant predictor of HA concentration in the previously referenced series of 8 dogs with septic peritonitis [14]. Elevated IL-6 has also been reported in a recent publication in dogs with organ dysfunction secondary to sepsis and evidence of EG degradation [16]. The subsequent lack of association between IL-6 and HA concentrations during our study period (at times after T0), may be caused by a predominant effect of cumulative fluid volumes, masking the effect of IL-6. The effect of inflammation on the EG might also have been attenuated by treatments aiming at controlling the source of inflammation and reversing shock.

Atrial natriuretic peptide is a protein secreted by the atrial myocytes in response to wall stretch, which promotes natriuresis and vasodilation [71]. It has also been shown to increase capillary permeability, which has been hypothesized to be mediated by EG degradation [41]. This hypothesis was supported by two experimental studies on guinea pigs [41,42] and a few human studies [32,44,72]. However, other studies in pigs, dogs, and people have found no correlation between ANP and EG degradation [15,29,31,39,61]. A true association between ANP and HA therefore remains uncertain. In our study, ANP did not have a significant effect on HA concentration. The concentration of ANP was also not significantly different between disease groups and not correlated with cumulative fluid volume in our groups, which could indicate that volume loading was possibly not achieved despite administration of IV fluids in our study (in the face of hemorrhage and vascular leak). Moreover, although the ANP assay used has been validated for dogs and previously used in a canine study [15] and the manufacturer’s instructions were followed, the coefficient of variability for ANP was moderately high in our study (mean + /- SD: 13.8% + /- 14.1), which could have led to inaccurate results. Therefore, our ANP results should be interpreted with caution.

Interestingly, in this canine study, no major increase in HA concentration was noted between T2 and T6, which is when the vast majority of patients underwent surgery. This finding is in agreement with previous human studies showing an increase in syndecan-1 was much less pronounced with surgery (by 1.3-1.5-fold) [72–75] than with sepsis and trauma (up to 20-fold) [12,20,56].

In this study, the illness severity score APPLE_fast_ had a significant effect on HA concentration, which could suggest that the severity of disease and endotheliopathy are associated, although causality is not established. The concentration of HA has been associated with disease severity in multiple human studies [12,23,76,77]. However, in our study, HA concentration was not associated with patient outcome. The concentration of HA on presentation and the progression of HA during hospitalization has been associated with outcome in people with sepsis [12,52,76,78] and trauma [23,69]. Since the overall mortality rate in our study was low (15%), it is possible that significant predictors of outcome could not be identified. Other studies have also failed to find an association between EG biomarkers and outcome [77].

Our study had several limitations. Due to its observational nature, treatments were not standardized, and several factors changed concomitantly, including patient signalment, underlying disease, disease severity, volume and type of intravenous fluids administered, as well as other interventions (e.g., surgery and administration of IV fluids prior to referral/ study enrolment). By using a multivariate model, we tried to account for as many parameters as possible, however some could not be included and might have affected the results. Patient subgroups were too small to investigate the individual effects of these factors on HA results. Although disease severity was heterogeneous, our patient population was overall not very critically ill, with an overall mortality rate of 15%, and mean + /- SD APPLE_full_ score of 33.8 + /- 10.1 out of 80 and APPLE_fast_ score of 26.4 + /- 6.9 out of 50. The applicability of these results to more critically ill patients is unknown. In addition, we did not include a control group to compare HA concentrations in sick dogs with healthy dogs and dogs who did not receive IV fluids, however patients acted as their own controls. Some patients had comorbidities which are known to affect HA concentrations, such as malignancy, liver disease, and hyperglycemia [79–82]. While malignancy was included in our multivariate model and was found not to have a significant effect on HA concentration, an insufficient number of dogs had liver disease and hyperglycemia to include these factors in the model, and they could have affected HA concentrations [80,81]. However, in our study only one patient had underlying liver disease and 8 patients had mild-moderate hyperglycemia (8−16 mmol/L) which was limited to 1 or 2 time points. Other limitations unfortunately include that the individual effect of fluid boluses, hypertonic saline, and transfusions could not be assessed due to the small number of patients that received each of these interventions. This should be investigated in a study designed for this purpose. In addition, the potential effects of hemodilution on HA concentrations were not explored. Hemodilution may have caused a decrease in measured HA concentration. The measured HA concentration could have been normalized to albumin concentration or hematocrit to address this, however these parameters are also likely to change due to hemorrhage and critical illness and not only hemodilution. The effect of hemodilution would be expected to underestimate the increase in HA concentration induced by cumulative volume of IV fluids, and more significant differences might have been observed without the effect of hemodilution. Finally, the study only investigated HA as a single biomarker of EG degradation, and other biomarkers of endothelial damage such as syndecan-1, heparan sulfate, VE-cadherin, vascular endothelial growth factor, plasminogen activator inhibitor-1, and von Willebrand factor might have led to different results [16,57]. Validation of assays to measure other EG biomarkers (syndecan-1, heparan sulfate) in dogs is warranted and multiple biomarkers should be used in future studies.

## Conclusion

In this study, HA concentrations were not significantly different between patients with HEM, NPS, and PS at different predetermined time points. The study did however establish that administration of increasing cumulative IV fluid volumes is associated with EG degradation in critically ill dogs with sepsis and hemorrhagic shock. Patients with more severe inflammation based on elevated IL-6 concentrations also appear to be at higher risk of EG degradation. The results add to growing concerns about endothelial damage caused by liberal fluid resuscitation, particularly in patients with sepsis and hemorrhagic shock. Future research could include trials comparing the effects of different types of fluid strategies on EG biomarkers to better characterize the safest approach to fluid resuscitation. Beyond the cumulative volume of fluids, investigating the effect of fluid boluses, hypertonic saline, colloids, and transfusions specifically would be interesting.

## Supporting information

S1 Fig Patient characteristics. (XLSX)

S2 Fig Fluid and biomarkers data. (XLSX)
